# Body shape index: Sex-specific differences in predictive power for all-cause mortality in the Japanese population

**DOI:** 10.1371/journal.pone.0177779

**Published:** 2017-05-16

**Authors:** Yuji Sato, Shouichi Fujimoto, Tsuneo Konta, Kunitoshi Iseki, Toshiki Moriyama, Kunihiro Yamagata, Kazuhiko Tsuruya, Ichiei Narita, Masahide Kondo, Masato Kasahara, Yugo Shibagaki, Koichi Asahi, Tsuyoshi Watanabe

**Affiliations:** 1 Dialysis Division, University of Miyazaki Hospital, Miyazaki, Japan; 2 Department of Hemovascular Medicine and Artificial Organs, Faculty of Medicine, University of Miyazaki, Miyazaki, Japan; 3 Steering Committee for Research on “Design of the Comprehensive Health Care System for Chronic Kidney Disease Based on the Individual Risk Assessment by Specific Health Check”, Fukushima, Japan; The University of Tokyo, JAPAN

## Abstract

**Background:**

While body mass index (BMI) is the most widely used anthropometric measure, its association with all-cause mortality is generally J-shaped or U-shaped. A body shape index (ABSI) is a recently formulated anthropometric measure that shows linear relationship to all-cause mortality, especially in Caucasian cohorts. We aimed to address the relationship between ABSI and all-cause mortality in Asians and to assess the influence of sex difference and of chronic kidney disease (CKD) on this relationship.

**Methods:**

This was a longitudinal cohort study assessing the association of ABSI, BMI, waist circumference (WC), and waist-to-height ratio (WHtR) with all-cause mortality in a Japanese nationwide Specific Health Checkup database. The study enrolled 160,625 participants followed-up between 2008 and 2012. We calculated the all-cause mortality risk associated with a 1-standard deviation increase (+1SD) in ABSI, BMI, WC, or WHtR in cohorts stratified by sex and the presence of CKD.

**Results:**

During the 4-year follow up, 1.3% of participants died. In men, ABSI (+1SD) significantly increased the risk for all-cause mortality after adjusting for other known risk factors including CKD; hazard ratio (HR) and 95% confidence intervals (CI) of non-CKD cohort, 1.30 (1.18 to 1.43), p<0.01; HR and 95%CI of CKD cohort, 1.16 (1.01 to 1.34), p = 0.04. In women, ABSI (+1SD) did not show significant association with all-cause mortality, especially in the CKD cohort; HR and 95% CI of non-CKD cohort, 1.07 (0.99 to 1.17), p = 0.09; HR and 95%CI of CKD cohort, 0.98 (0.84 to 1.14), p = 0.78. Conversely, BMI (+1SD) was associated with significantly lower risk in men, although minimal association was found in women. WC and WHtR showed little association with all-cause mortality. On stratification per ABSI quartiles, mortality risk increased linearly and significantly with ABSI in men, but not in women with CKD. Both BMI and WC showed significant but U-shaped association with mortality in the non-CKD cohort and in men with CKD. WHtR also showed significant U-shaped association with mortality in men.

**Conclusions:**

In the Japanese population, ABSI showed significant and linear correlation with mortality risk in men but not in women, especially in the presence of CKD.

## Introduction

A body shape index (ABSI) was formulated based on the National Health and Nutrition Examination Survey (NHANES) cohort to serve as a predictive anthropometric measure for all-cause mortality [1]. ABSI is calculated based on body height (BH), body weight (BW), and waist circumference (WC). A high value of ABSI suggests relatively higher WC compared to BH and BW [1]. Dual-energy X-ray absorptiometry measurements indicated that ABSI is positively correlated with fat mass index and negatively correlated with fat-free mass index [2].

Body mass index (BMI) has been widely used to identify overweight or obese individuals so that appropriate measures can be taken to prevent the development of diabetes, hypertension, and dyslipidemia, with the ultimate goal of preventing cardiovascular events. Although, many epidemiologic studies that used BMI as a variable showed there is a U-shaped or J-shaped association between BMI and mortality risk not only in the Japanese population [3,4] but also in the Caucasian population [5–7], a proportional association was noted between ABSI and mortality risk [1, 8–12]. WC is thought to reflect central obesity and therefore is sometimes used as a complementary parameter of BMI; however, WC is sensitive to body size. Waist-to-height ratio (WHtR) was also reported to be a better marker than BMI for predicting mortality [13], but its usefulness in the clinical setting remains uncertain.

The presence of chronic kidney disease (CKD) affects relationships between anthropometric measures and mortality. In CKD patients, the effect of BMI on mortality is controversial. Some [14–16] reported lower BMI associated with mortality risk, however, others [17–19] showed no consistent effect. In the evaluation of ABSI, only one paper [20] reported that CKD stage G5D cancelled the effect of ABSI on mortality. Further, a recent report from Taiwan [21] showed a reverse-J shape relation between BMI and mortality in CKD males, but no consistent relationship was observed in CKD females. These relationships of ABSI have not been reported so far. Therefore, we assessed the specific influence of sex and chronic kidney disease (CKD) on the predictive value of ABSI for all-cause mortality.

## Methods

### a. Study design and population

**Fig 1:**
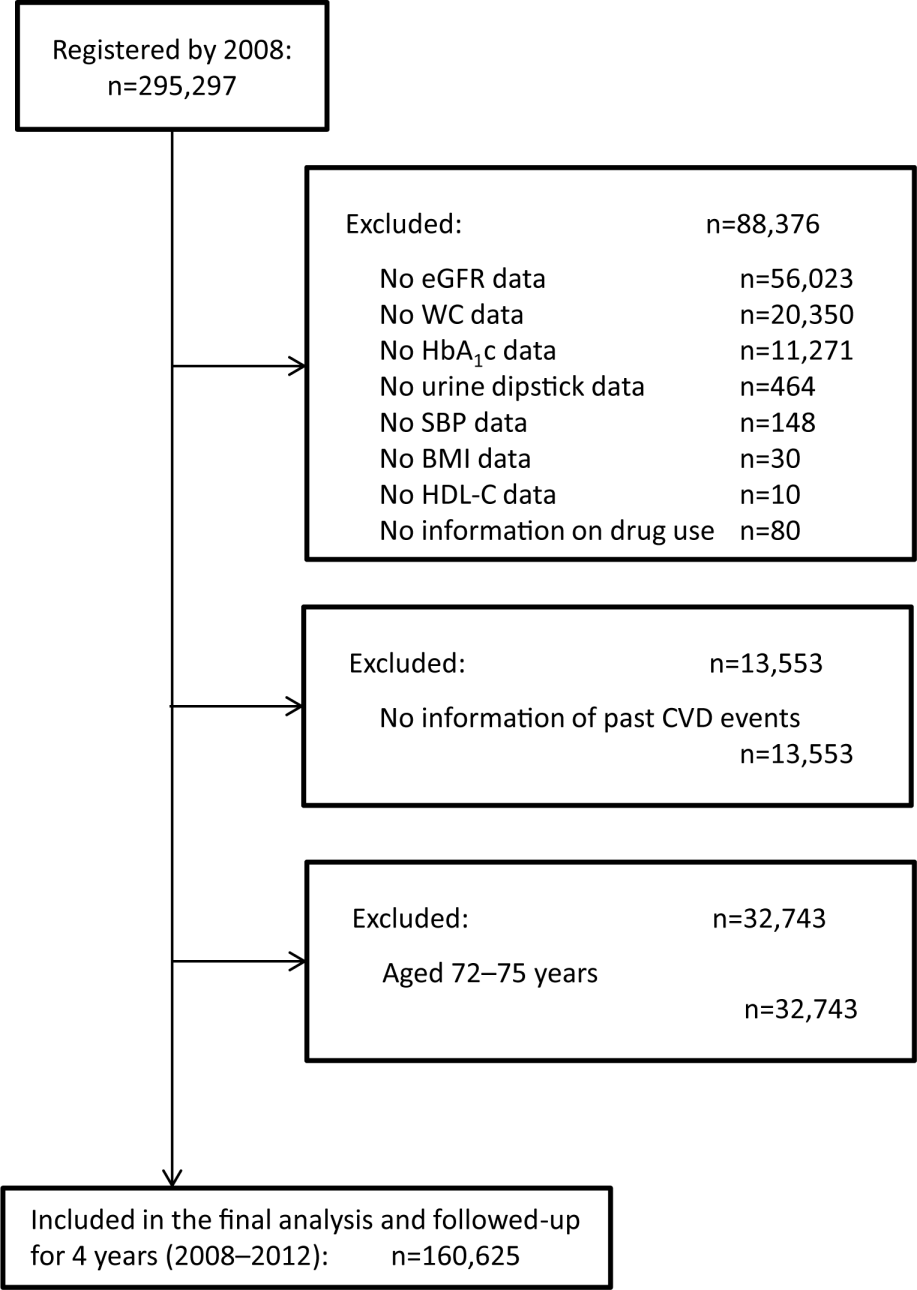
Flow chart of participant enrollment. Of the 295,297 individuals registered in the Specific Health Checkup program, 134,672 were excluded because of missing data or advanced age (>72 years), since the study was planned with a 4-year follow-up and the Specific Health Checkup program covered only individuals younger than 75 years. Finally, 160,625 individuals were included in our analysis. Abbreviations: BMI, body mass index; CVD, cardiovascular disease; eGFR, estimated glomerular filtration rate; HbA1c, glycated hemoglobin; HDL-C, high-density lipoprotein cholesterol; SBP, systolic blood pressure; WC, waist circumference.

In 2008, the Japanese government started a new annual health check program (the “Specific Health Checkup”) to support early diagnosis and intervention in metabolic syndrome. The target population comprises Japanese citizens aged 40–74 years and residing in different geographic regions [22–25]. A total of 295,297 individuals were registered in the annual health check program to be followed-up between 2008 and 2012 (Fig 1). Individuals with missing laboratory data were excluded. For example, 56,023, 11,271, and 464 individuals were excluded for missing data regarding the estimated glomerular filtration rate (eGFR), glycated hemoglobin (HbA_1_c) levels, and urine dipstick test results, respectively. Another 13,553 individuals were excluded for lacking information regarding past CVD events or because of advanced age (72–75 years), since the study involved a 4-year follow-up and the Specific Health Checkup program only covered individuals younger than 75 years. Finally, 160,625 individuals were included in the present analysis.

We performed an observational cohort study with all-cause mortality as the primary outcome measure. Information regarding death events occurring between 2008 and 2012 was obtained from the national death records. Further details regarding the methods applied in this study were previously described elsewhere [25]. This study was conducted according to the guidelines of the Declaration of Helsinki and was granted ethical approval by the ethics committee of Fukushima Medical University (No. 1485, 2771). Informed consent was not obtained from each participant because all data were anonymized before analysis.

### b. Baseline measurements

WC was measured at navel level, at the end of normal exhalation, with the participant standing upright. In participants with severe obesity, WC was measured at the mid distance between the lower edge of the rib cage and the superior anterior iliac spine. BH and BW were measured with the participant wearing light clothing but no shoes. BMI was calculated based on BH and BW (kg/m^2^).

Blood samples were obtained with the participants in fasting state. Urine dipstick test result was recorded as (-), (+/-), (1+), (2+), and (3+), with proteinuria defined at 1+ or more. The eGFR values were derived using the following equation specific to the Japanese population [26]:

$$eGFR(mL/\min/1.73m^{2})=194\times age{\left(years\right)}^{-0.287}\times serum\;creatinine{\left(mg/dL\right)}^{-1.094}\;(\times 0.739\;if\;female)$$

Systolic blood pressure (SBP) was measured using a standard sphygmomanometer or an automated device on the right arm after the participant had rested for 5 minutes in a seated position. CKD was defined as eGFR<60 mL/min/1.73 m^2^ or having proteinuria (1+ and more in the dipstick test). All subjects completed a questionnaire to document their medical history and current medications. Brain attacks included brain infarction and hemorrhage. Cardiac events included myocardial infarction and cardiac angina.

### c. Statistical analysis

All statistical analyses were performed using SPSS version 20.0J (SPSS Inc., Chicago, IL), except for the calculations involving Harrell’s C-index, for which the differences between models were analyzed according to a previously described protocol [27] using STATA/MP version 14 (StataCorp, College Station, TX). Data are expressed as mean ± standard deviation (SD). The study sample was stratified based on sex and the presence of CKD. Clinical and metabolic data were compared using the Kruskal-Wallis test, while categorical data were compared using the chi-squared test. Correlation coefficients describing the relationship among anthropometric parameters were computed using Spearman’s or Pearson’s method, as appropriate. Subsequently, Cox regression analyses were performed to examine the independent association of anthropometric parameters with all-cause mortality. In the multivariable analysis, these associations were assessed after adjustments for age and sex (model 1), as well as for SBP, high-density lipoprotein cholesterol (HDL-C) levels, HbA1c levels, eGFR, use of drugs (against hypertension, diabetes, and dyslipidemia), past history of CVD, and current smoking status (model 2). Statistical significance was defined as *P*<0.05.

Finally, the sex-specific effect of CKD on mortality was assessed using C-statistics. Model A included adjustment for ABSI (+1SD), age (+1SD), SBP (+1SD), HDL-C levels (+1SD), HbA1c levels (+1SD), drug use status (anti-hypertensive, anti-diabetes, or anti-dyslipidemia drug user), past CVD history, and smoking status (smoker). Another model was created after entering CKD into model A (model A+CKD). The predictive power for all-cause mortality was verified for each model, separately in men and in women.

## Results

### a. Sex-specific histograms of anthropometry parameters

The sex-specific distribution of values for each anthropometric parameter (S1 Fig) is approximately normal. However, differences were noted between men and women for each parameter.

### b. Age-specific changes in anthropometric parameters

**Fig 2:**
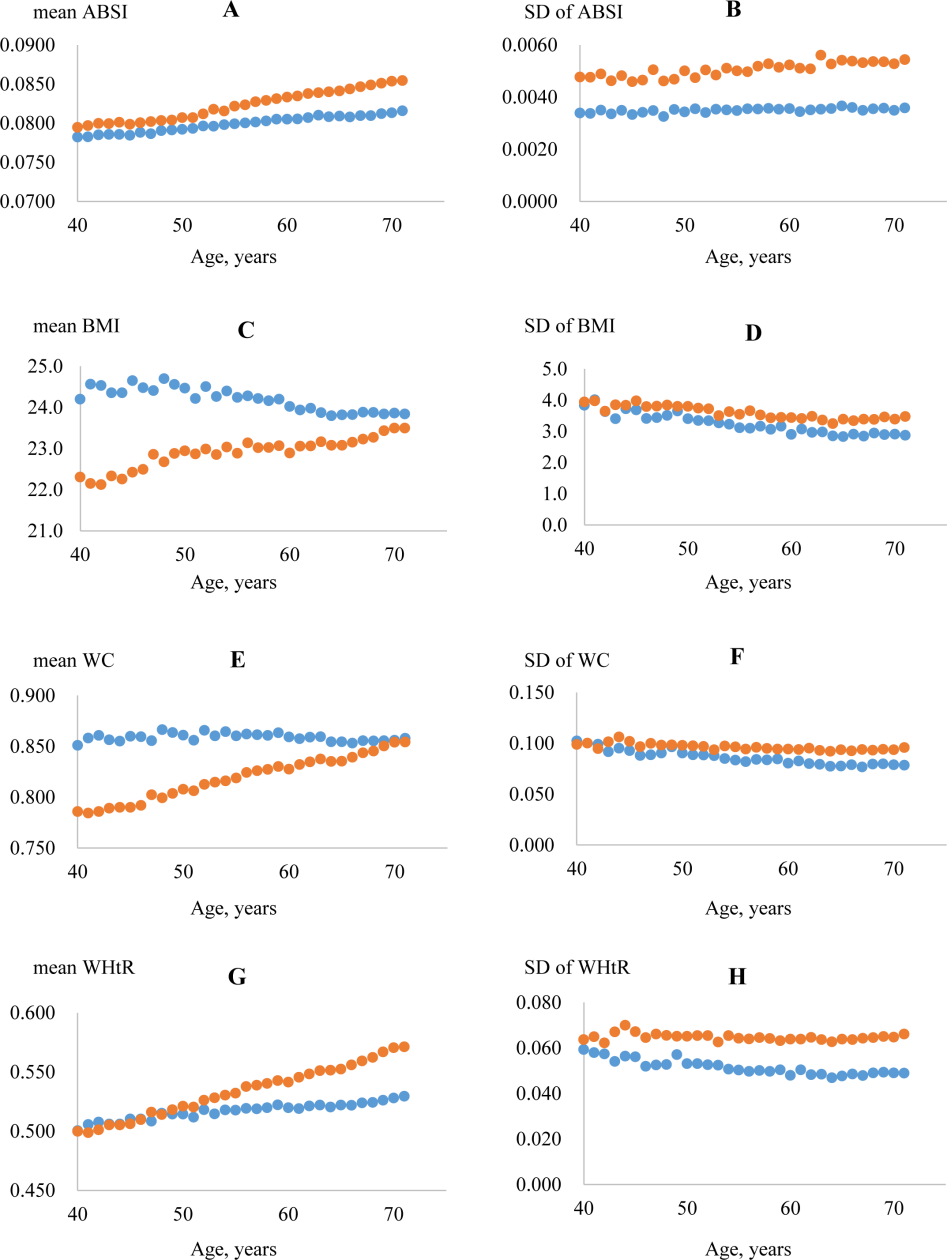
Age-specific changes in the values of anthropometric parameters. The analysis was performed separately in men (blue) and women (red). ABSI (A and B) and WHtR (G and H) increase linearly with age, particularly in women. On the other hand, BMI (C and D) and WC (E and F) decrease linearly or remain unchanged with age in men, whereas they increase linearly with age in women. Abbreviations: ABSI, a body shape index; BMI, body mass index; WC, waist circumference; WHtR, waist-to-height ratio.

ABSI and WHtR increased linearly with age, particularly in women (Fig 2A and 2B; 2G and 2H; respectively). In contrast, BMI and WC decreased linearly or remained constant with age in men, whereas they increased linearly with age in women (Fig 2C and 2D; 2E and 2F; respectively). As sex-specific differences were noted for each parameter, further analyses were necessarily adjusted for age and sex.

### c. Relevance of sex and CKD

The participants were stratified according to presence of CKD (Table 1). The prevalence of CKD was 17.4%. Regarding the overall distribution of values of anthropometric parameters, the effect of sex-based stratification was stronger than that of CKD-based stratification. Inter-group differences were also noted for other baseline parameters (e.g., SBP, HDL-C levels, HbA1c levels). All-cause mortality during the 4-year follow-up was higher in the CKD cohort; mortality was also higher in men than in women. Participants’ characteristics also divided by ABSI quartile in men and women were shown in S Tables 1 and 2, respectively. In men, as ABSI increased, SBP, A1c, and mortality rate increased, however, in women this relationship was blunted. Past history of CVD also paralleled with ABSI in both sexes; however, current smoking was not constant in both sexes.

**Table 1:** Characteristics at enrollment.

		non-CKD cohort	CKD cohort	*p* value
Number, n (%)		132,647 (82.6%)	27,978 (17.4%)	
Age, years old		61.1 (8.1)	64.1 (6.4)	<0.01
Sex, women		61.0%	47.4%	<0.01
ABSI	Men	0.0808 (0.0036)	0.0807 (0.0036)	<0.01
	Women	0.0835 (0.0055)	0.0837 (0.0053)	0.02
BMI, kg/m^2^	Men	23.9 (3.1)	24.6 (3.1)	<0.01
	Women	23.0 (3.4)	23.8 (3.8)	<0.01
WC, m	Men	0.85 (0.08)	0.87 (0.08)	<0.01
	Women	0.83 (0.10)	0.85 (0.10)	<0.01
WHtR	Men	0.52 (0.05)	0.53 (0.05)	<0.01
	Women	0.55 (0.07)	0.56 (0.07)	<0.01
BH, m	Men	1.65 (0.06)	1.64 (0.06)	<0.01
	Women	1.52 (0.06)	1.52 (0.06)	0.10
BW, kg	Men	65.1 (9.7)	66.3 (9.5)	<0.01
	Women	53.2 (8.3)	55.0 (9.0)	<0.01
SBP, mmHg		128 (17)	132 (18)	<0.01
HDL-C, mg/dL		61.5 (15.6)	58.3 (15.5)	<0.01
HbA1c, %		5.33 (0.68)	5.45 (0.85)	<0.01
eGFR, mL/min/1.73m^2^		79.1 (13.9)	58.6 (14.5)	<0.01
Anti-hypertensive drug		23.2%	38.2%	<0.01
Anti-diabetes drug		4.2%	7.5%	<0.01
Anti-dyslipidemic drug		12.6%	17.6%	<0.01
Past history of CVD		6.7%	11.9%	<0.01
Current smoking		14.9%	13.4%	<0.01
Urine dipstick test				<0.01
	-	92.1%	63.0%	
	±	7.9%	6.8%	
	1+	0.0%	20.5%	
	2+	0.0%	7.3%	
	3+	0.0%	2.3%	

Data given as mean (standard deviation) unless otherwise specified. *P*-values refer to the differences between the CKD and non-CKD cohorts.

Abbreviations: CKD, chronic kidney disease; ABSI, a body shape index; BMI, body mass index; WC, waist circumference; WHtR, waist-to-height ratio; BH, body height; BW, body weight; SBP, systolic blood pressure; HDL-C, high-density lipoprotein cholesterol; HbA1c, glycated hemoglobin; eGFR, estimated glomerular filtration rate; CVD, cardiovascular disease.

**Table 2:** Risk for all-cause mortality for each 1SD increment in the value of relevant anthropomorphic parameters.

		Men				Women			
		non-CKD cohort		CKD cohort		non-CKD cohort		CKD cohort	
		HR (95%CI)	*P*-value	HR (95%CI)	*P*-value	HR (95%CI)	*P*-value	HR (95%CI)	*P*-value
ABSI, +1SD	Model 1	1.38 (1.26 to 1.52)	<0.01	1.23 (1.07 to 1.42)	<0.01	1.09 (1.00 to 1.18)	0.049	1.00 (0.87 to 1.16)	0.97
	Model 2	1.30 (1.18 to 1.43)	<0.01	1.16 (1.01 to 1.34)	0.04	1.07 (0.99 to 1.17)	0.09	0.98 (0.84 to 1.14)	0.78
BMI, +1SD	Model 1	0.90 (0.83 to 0.97)	<0.01	0.87 (0.78 to 0.98)	0.02	0.97 (0.89 to 1.06)	0.53	1.15 (1.02 to 1.31)	0.03
	Model 2	0.87 (0.80 to 0.94)	<0.01	0.84 (0.74 to 0.95)	0.01	0.91 (0.83 to 1.00)	0.047	1.03 (0.90 to 1.19)	0.66
WC, +1SD	Model 1	1.02 (0.94 to 1.10)	0.64	0.93 (0.83 to 1.05)	0.22	1.01 (0.93 to 1.11)	0.75	1.13 (0.98 to 1.29)	0.09
	Model 2	0.98 (0.90 to 1.06)	0.59	0.88 (0.78 to 1.00)	0.045	0.96 (0.88 to 1.05)	0.38	1.00 (0.86 to 1.16)	0.99
WHtR, +1SD	Model 1	1.02 (0.94 to 1.11)	0.59	0.98 (0.86 to 1.10)	0.70	1.03 (0.95 to 1.12)	0.43	1.15 (1.01 to 1.31)	0.04
	Model 2	0.98 (0.90 to 1.07)	0.59	0.93 (0.82 to 1.06)	0.27	0.98 (0.90 to 1.07)	0.67	1.03 (0.90 to 1.19)	0.67

Risk was assessed using Cox regression analysis. Model 1 was adjusted for age and sex. Model 2 consisted of Model 1 + adjustments for systolic blood pressure (+1SD), high-density lipoprotein cholesterol (+1SD), glycated hemoglobin (+1SD), estimated glomerular filtration rate (+1SD), drug use (anti-hypertension, anti-diabetes, or anti-dyslipidemia), past history of cardiovascular disease, and current smoking status (smoker).

Data given as hazard ratio (HR) with 95% confidence interval (95%CI).

Abbreviations: ABSI, a body shape index; BMI, body mass index; CKD, chronic kidney disease; SD, standard deviation; WC, waist circumference; WHtR, waist-to-height ratio.

### d. Correlation of anthropometric parameters according to sex and presence of CKD

Pairwise correlation coefficients for ABSI, BMI, WC, WHtR, BH, and BW were computed after stratification according to sex and presence of CKD (S1–S4 Tables). Similar patterns were observed for all groups. Specifically, ABSI did not correlate with BMI, BH, or BW, and showed only modest correlation with WC and WHtR, suggesting that ABSI is independent from BMI. BMI correlated strongly with WC, WHtR, and BW, but not with BH.

### e. Risk factors for all-cause mortality

During the follow-up, 1.0% and 1.9% of participants died in non-CKD and CKD cohort, respectively. Hazard ratios for all-cause mortality were calculated per 1-SD increase in each anthropometric parameter (Table 2). In men with or without CKD, the 1-SD increase in ABSI was associated with a significant increase in mortality risk both according to Model 1 (adjusted for age and sex) and according to Model 2 (adjusted for age, sex, SBP, HDL-C levels, HbA1c levels, eGFR, anti-hypertensive drug use, anti-diabetic drug use, anti-hyperlipidemic drug use, past history of cardiovascular disease, and current smoking status); fully adjusted hazard ratio (HR) and 95% confidence intervals (CI) of non-CKD cohort, 1.30 (1.18 to 1.43), p<0.01; CKD cohort, 1.16 (1.01 to 1.34), p<0.04. In women, ABSI did not show significant association with all-cause mortality, especially in the CKD cohort; fully adjusted HR and 95% CI of non-CKD cohort, 1.07 (0.99 to 1.17), p = 0.09; CKD cohort, 0.98 (0.84 to 1.14), p = 0.78. Risk of covariates other than ABSI was shown in S7 Table. Age, SBP, past CVD history, and current smoking were positively and significantly associated with all-cause mortality. Oddly, eGFR was positively correlated with mortality in subjects with normal eGFR level (≧60 mL/min/1.73m^2^). Conversely, increased BMI was associated with significantly lower risk in men, but no consistent association was noted in women. WC and WHtR also showed no consistent association with all-cause mortality.

Subsequently, each group was further stratified into quartiles based on the distribution of values for each anthropometric parameter evaluated. HRs for all-cause mortality was calculated considering the first quartile as reference (Table 3and S8 Table). Both Model 1 and Model 2 indicated that the hazard ratios describing the association between ABSI and mortality risk increased significantly in a stepwise fashion in both men and women without CKD, as well as in men with CKD, but not in women with CKD. In contrast, BMI and WC showed a significant but U-shaped association with mortality in the non-CKD cohort and in men with CKD. WHtR showed no significant association with mortality.

**Table 3:** ABSI quartile for all-cause mortality in participants with and without chronic kidney disease.

			Q1	Q2		Q3		Q4	
				HR (95%CI)	*P*-value	HR (95%CI)	*P*-value	HR (95%CI)	*P*-value
Men	non-CKD	Model 1	1.00 (Ref)	1.06 (0.85 to 1.32)	0.61	1.39 (1.13 to 1.71)	0.01	1.65 (1.35 to 2.01)	<0.01
		Model 2	1.00 (Ref)	1.02 (0.82 to 1.27)	0.87	1.30 (1.06 to 1.60)	0.01	1.48 (1.21 to 1.80)	<0.01
	CKD	Model 1	1.00 (Ref)	0.86 (0.62 to 1.19)	0.35	1.02 (0.75 to 1.39)	0.91	1.47 (1.10 to 1.96)	0.01
		Model 2	1.00 (Ref)	0.84 (0.61 to 1.17)	0.31	0.97 (0.71 to 1.32)	0.85	1.34 (1.01 to 1.79)	0.046
Women	non-CKD	Model 1	1.00 (Ref)	1.15 (0.87 to 1.52)	0.32	1.34 (1.03 to 1.75)	0.03	1.31 (1.00 to 1.71)	0.049
		Model 2	1.00 (Ref)	1.13 (0.86 to 1.49)	0.38	1.31 (1.00 to 1.71)	0.047	1.27 (0.97 to 1.66)	0.08
	CKD	Model 1	1.00 (Ref)	1.10 (0.70 to 1.71)	0.69	1.00 (0.64 to 1.57)	0.99	1.12 (0.71 to 1.75)	0.63
		Model 2	1.00 (Ref)	1.05 (0.67 to 1.64)	0.85	0.93 (0.60 to 1.47)	0.77	1.05 (0.67 to 1.65)	0.82

Risk was assessed using Cox regression analysis. Model 1 was adjusted for age. Model 2 consisted of Model 1 + adjustments for systolic blood pressure (+1SD), high-density lipoprotein cholesterol (+1SD), glycated hemoglobin (+1SD), estimated glomerular filtration rate (+1SD), drug use (anti-hypertension, anti-diabetes, or anti-dyslipidemia), past history of cardiovascular disease, and current smoking status (smoker).

Data given as hazard ratio (HR) with 95% confidence interval (95%CI) and stratified by quartiles (Q1 to Q4) for the distribution of values for each anthropometric parameter.

Abbreviations: ABSI, a body shape index; CKD, chronic kidney disease; Ref, reference.

To evaluate the validity of Cox proportional model, the log minus log function at mean of ABSI quartile was calculated. Graphs indicated that proportional hazard assumption applied in men (S2A Fig) but not in women (S2B Fig).

### f. Correlation between WC and BW^2/3^×BH^-5/6^

According to the original report describing ABSI [1], the predictive capability of ABSI stems mainly from the high and positive correlation between WC and BW^2/3^ × BH^-5/6^. In other words, the difference in ABSI reflects the difference in WC given BW and BH. Indeed, we found very high and positive correlation (*r* = 0.837) between WC and BW^2/3^ × BH^-5/6^ in our Japanese cohort. In men and women, the correlation coefficients were 0.884 and 0.829, respectively. Further stratification according to the presence of CKD did not affect these correlations.

### g. Effect of sex and CKD on the discriminative ability of ABSI

We examined the C-index for all-cause mortality in men and women using Model A, which included only ABSI, and Model A+CKD, which included ABSI and CKD (Table 4). In men, Model A had significant predictive power for mortality, and adding CKD did not improve this effect (*P* = 0.56). On the other hand, in women, adding CKD improved predictive power significantly (*P* = 0.02).

**Table 4:** Sex-specific differences in C-indexes regarding predictive power for all-cause mortality.

		C-index	*P*-value	*P*-value between models
Men	Model A	0. 65 (0.62 to 0.67)	<0.01	0.56
	Model A+CKD	0.64 (0.62 to 0.67)	<0.01	
Women	Model A	0.60 (0.57 to 0.63)	<0.01	0.02
	Model A+CKD	0. 62 (0.59 to 0.65)	<0.01	

Discriminating power was assessed using C-statistics.

Model A was adjusted for a body shape index (+1SD), age (+1SD), systolic blood pressure (+1SD), high-density lipoprotein cholesterol (+1SD), glycated hemoglobin (+1SD), estimated glomerular filtration rate (+1SD), drug use (anti-hypertension, anti-diabetes, or anti-dyslipidemia), past history of cardiovascular disease, and current smoking status (smoker).

Abbreviations: CKD, chronic kidney disease; SD, standard deviation

## Discussion

Our main finding is that the new anthropometric measure ABSI can be applied to predict all-cause mortality in Japanese men, but it shows only weak association with all-cause mortality in Japanese women, especially in the presence of CKD. ABSI was originally developed based on the NHANES cohort, which mainly consisted of white, black, and Hispanic participants [1]. If ABSI is defined as WC / (BMI^2/3^×BH^1/2^), higher ABSI implies relatively higher WC given similar BH and BW, since WC is highly proportional to BW^2/3^×BH^-5/6^ [1].

To date, 17 epidemiological studies using ABSI have been published. Overall, ASBI showed superior predictive power for mortality or morbidity in Caucasians than in Asians. Five investigations assessed all-cause mortality as an endpoint. Of these, three reports based on data from Caucasian participants indicated that ABSI could predict mortality [1, 9, 28], while one paper failed to demonstrate the significance of ABSI in Iranian women without exploring this finding [29]. Another study, which enrolled dialysis patients and was the only study to consider CKD patients, also reported no significance of ABSI in the Turkish population [20]. Three studies assessing all-cause mortality and CVD death indicated that ABSI had predictive power in European populations [8, 10, 12]. Another nine studies enrolled European, Iranian, Indonesian, Chinese, and Japanese participants but provided conflicting results regarding the significance of ABSI for detecting or predicting comorbidities such as diabetes, hypertension, and dyslipidemia [30–38].

Taken together, the findings of these previous studies indicate that there are race-specific differences in the predictive power of ABSI, which was originally formulated based on data from white, black, and Hispanic individuals [1]. It assumed that there are some anthropometric differences between Caucasians and Asians. For example, Asians are relatively shorter than Caucasians. In addition, WC measurement approaches vary. Specifically, in the original study that described ABSI, WC was measured at the iliac crest [1], whereas studies from Japan and China involved WC measured at the umbilicus level [30, 32, 33]. Another study from Indonesia did not describe the WC measuring method [35].

In addition to racial differences, significant sex-specific differences were noted in our study, as well as in past reports [28, 36, 37]. Specifically, we found that the predictive power of ABSI for mortality was not evident in women with CKD, whereas some significance was noted in women without CKD. There are some speculations why ABSI may not be a good predictor for mortality or morbidity in women. First, for women, the mortality rates in our Japanese cohort were relatively lower than those noted in Caucasian cohorts [1, 9, 27]. The small number of deaths among the women in our cohort might have decreased the statistical power for the mortality analysis. Second, the original paper indicated that ABSI is higher in men than in women [1], whereas our data indicated the opposite, which might also be related to race-specific differences. Third, the correlation between WC and BW^2/3^×BH^-5/6^ was not as good in our female cohort, which is relevant because high correlation between these measures forms the basis of the predictive power of ABSI. Fourth, fat distribution differs by sex, with more central fat deposition in women, and could be one reason that the effect of ABSI on mortality was not seen [2,39].

The impact of CKD on mortality may be sex-specific. Our C-statistics showed that presence of CKD in women increased the predictive power of ABSI, but this phenomenon was not observed in men. A similar effect was also observed in a survey of a cohort based on the general Japanese population [40], which indicated that the presence of CKD and other covariates similar to those included in our study was significantly correlated to cardiovascular events in women. The reason women experience a more pronounced effect of CKD in terms of morbidity or mortality remains unclear.

Risk for all-cause mortality of covariates when fully adjusted Cox analysis, age, SBP, past CVD history, and current smoking were almost significantly and positively associated. However, increase of eGFR of subjects whose eGFR 60 mL/min/1.73m^2^ and more also significantly and positively associated with all-cause mortality. This phenomenon was previously reported in a meta-analysis of general population cohorts [41]. Higher eGFR values did not correlate well with inulin clearance [26]. Therefore, higher eGFR values need to be interpreted carefully.

Three are some limitations in our study. First, information of chronic preexisting conditions except past CVD, such as chronic obstructive pulmonary disease or malignancy, is unavailable and is often associated with low body weight or low BMI. The health checkup questionnaire did not request this information. Further, smoking status information was collected only as current smoking or not, so past smoking history was unavailable. Second, we could not exclude the possibility that the statistical power of analysis was affected by the relatively low incidence of death (compared to that noted in the original US cohort, especially for women) during the 4-year follow-up. Further studies are warranted to clarify whether ABSI is suitable for mortality prediction in Japanese women. Finally, it is difficult to perform a direct comparison between the results from different countries given the variability in the methods of measuring WC.

Despite its limitations, the present study brought evidence that ABSI has significant and linear association with mortality risk in Japanese men but not in women, especially in the presence of CKD. Our findings regarding ABSI are relevant since predicting mortality based on BMI is difficult, as many reports have described a U-shaped or J-shaped relationship between BMI and mortality [3–7].

## Supporting information

S1 Fig Sex-specific histograms of anthropometry parameters. For each anthropometric parameter, the distribution of values is almost normal. Sex-specific differences in the distribution of values is for ABSI (A: men; B: women), BMI (C: men; D: women), WC (E: men; F: women), and WHtR (G: men; H: women).
(PDF)

S2 Fig Log minus log function at mean of covariates. (PDF)

S1 Table Characteristics at enrollment and all-cause mortality over the 4-year follow-up divided by quartile of a body shape index in men. (DOCX)

S2 Table Characteristics at enrollment and all-cause mortality over the 4-year follow-up divided by quartile of a body shape index in women. (DOCX)

S3 Table Correlation between anthropometric parameters in men without chronic kidney disease. (DOCX)

S4 Table Correlation between anthropometric parameters in women without chronic kidney disease. (DOCX)

S5 Table Correlation between anthropometric parameters in men with chronic kidney disease. (DOCX)

S6 Table Correlation between anthropometric parameters in women with chronic kidney disease. (DOCX)

S7 Table Hazard ratios and 95% confidence intervals of other covariates when risk of 1-SD increase of ABSI for all-cause mortality was analyzed by Cox analysis. (DOCX)

S8 Table Risk for all-cause mortality in participants with and without chronic kidney disease. (DOCX)
