# Supplementary material for: Body shape index: Sex-specific differences in predictive power for all-cause mortality in the Japanese population
Source: PLoS One. 2017 May 16;12(5):e0177779. doi: 10.1371/journal.pone.0177779 (PMC5433760; doi:10.1371/journal.pone.0177779)
Supplement: S1 Fig — For each anthropometric parameter, the distribution of values is almost normal. Sex-specific differences in the distribution of values is for ABSI (A: men; B: women), BMI (C: men; D: women), WC (E: men; F: women), and WHtR (G: men; H: women). (PDF) [file pone.0177779.s001.pdf]

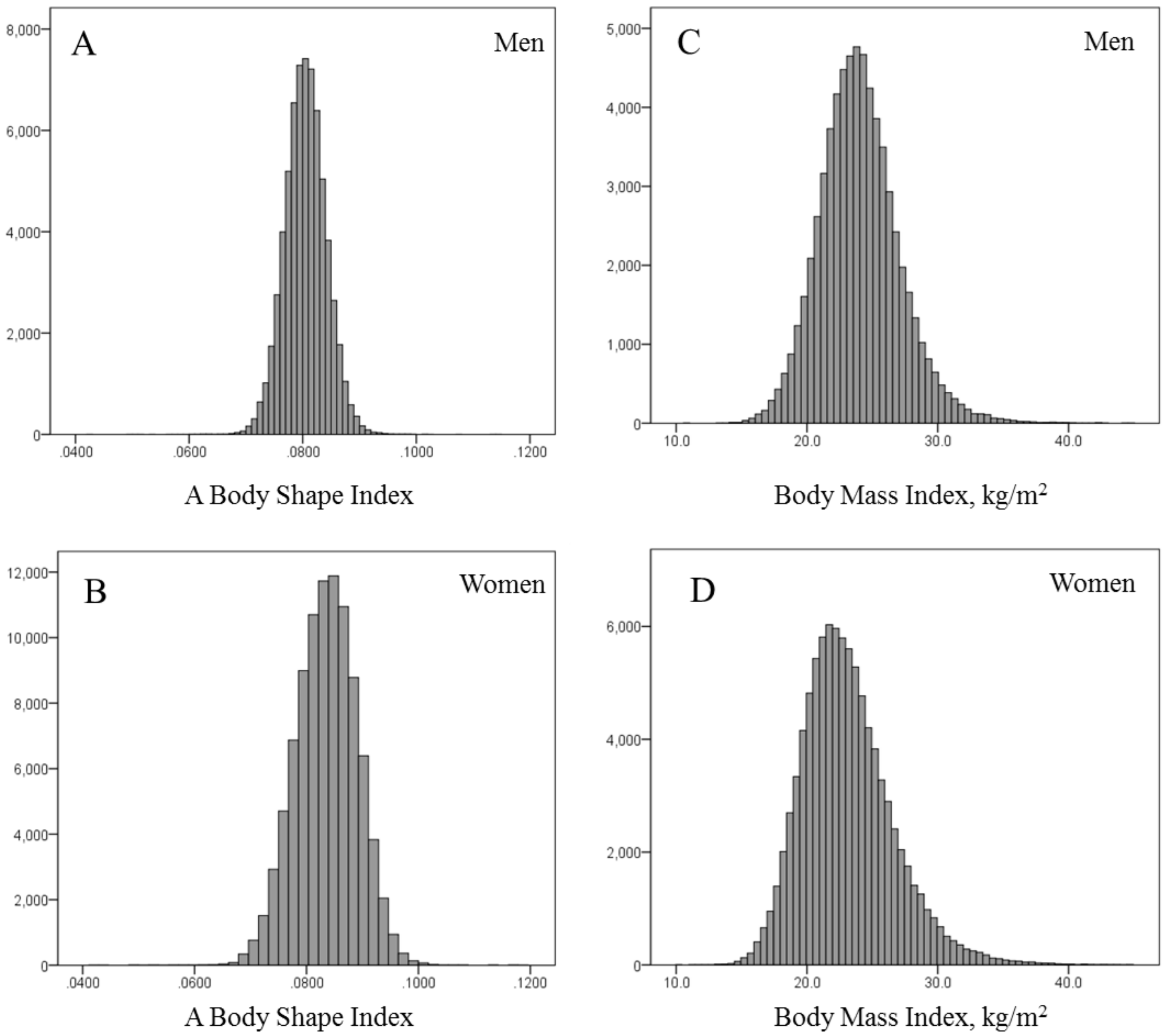
A
Men
8,000
6,000
4,000
2,000
0
.0400
.0600
.0800
.1000
.1200
A Body Shape Index
C
Men
5,000
4,000
3,000
2,000
1,000
0
10.0
20.0
30.0
40.0
Body Mass Index, kg/m2
B
Women
12,000
10,000
8,000
6,000
4,000
2,000
0
.0400
.0600
.0800
.1000
.1200
A Body Shape Index
D
Women
6,000
4,000
2,000
0
10.0
20.0
30.0
40.0
Body Mass Index, kg/m2

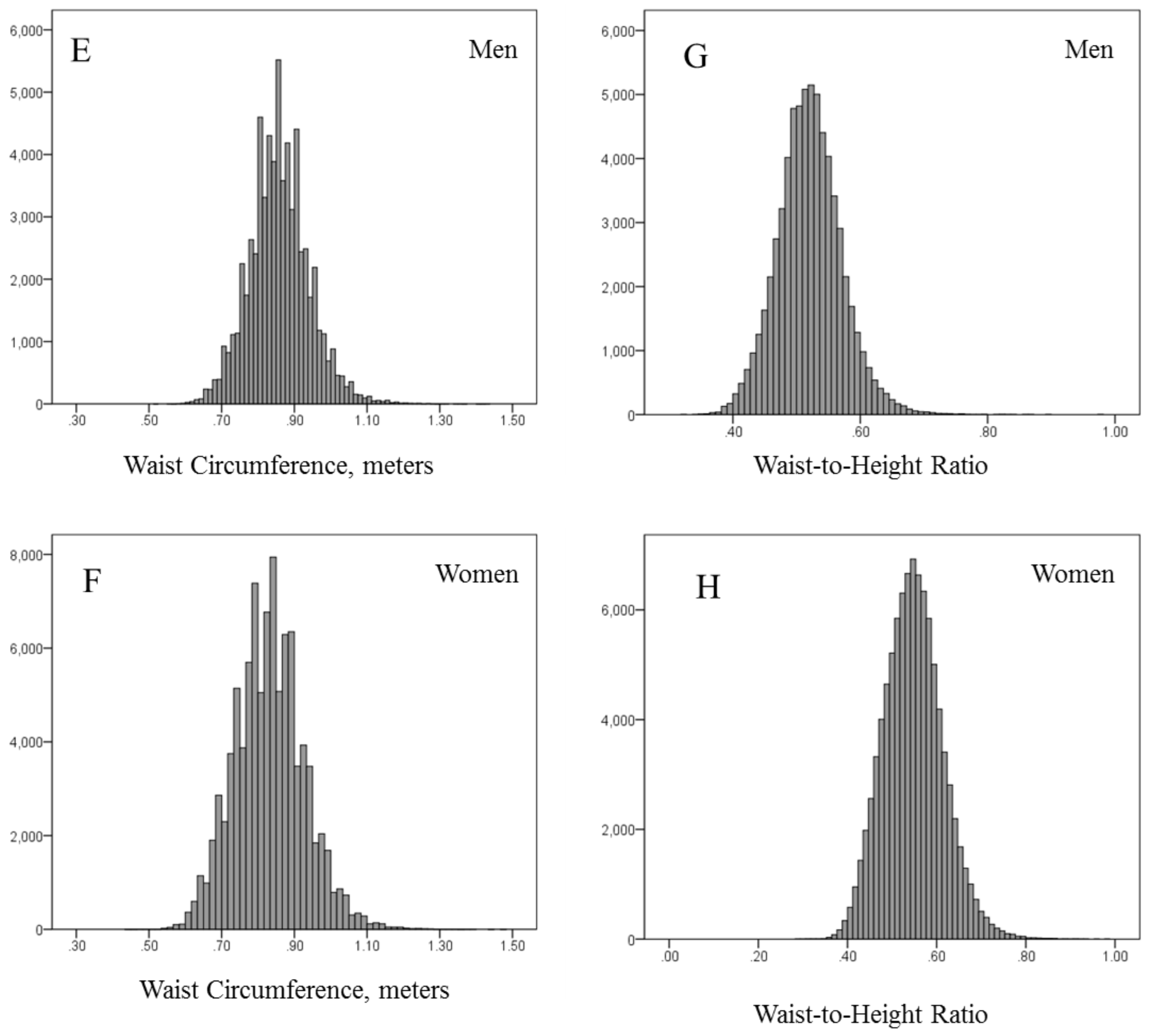
E
Men
Waist Circumference, meters
G
Men
Waist-to-Height Ratio
F
Women
Waist Circumference, meters
H
Women
Waist-to-Height Ratio
