# Supplementary figures and images for: Body shape index: Sex-specific differences in predictive power for all-cause mortality in the Japanese population
Source: PLoS One. 2017 May 16;12(5):e0177779. doi: 10.1371/journal.pone.0177779 (PMC5433760; doi:10.1371/journal.pone.0177779)

**A**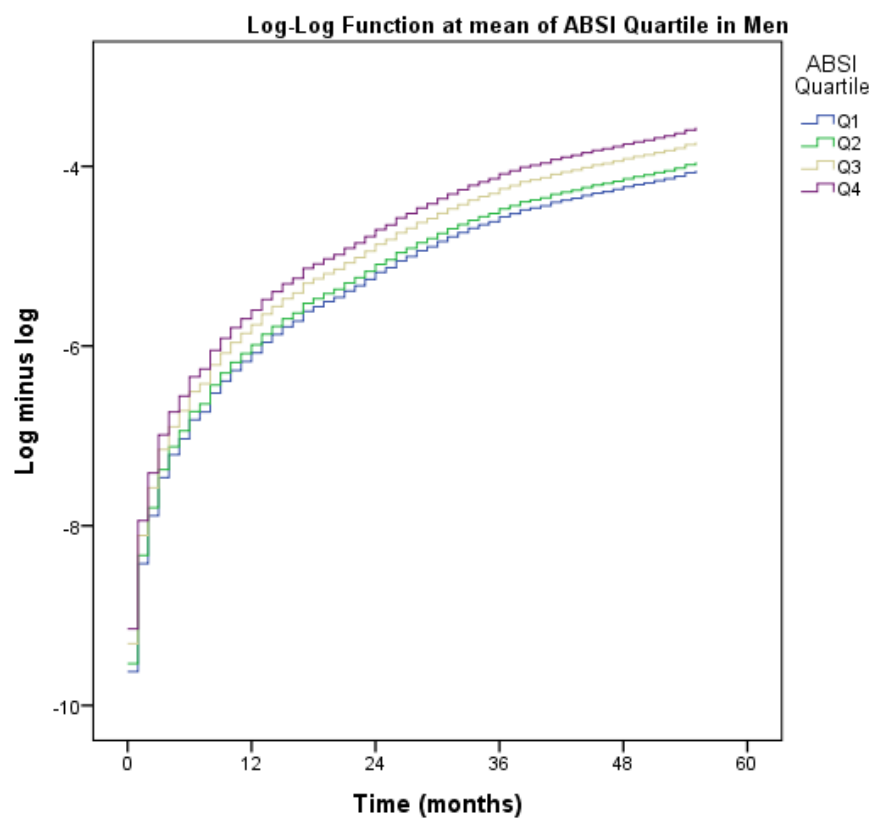**B**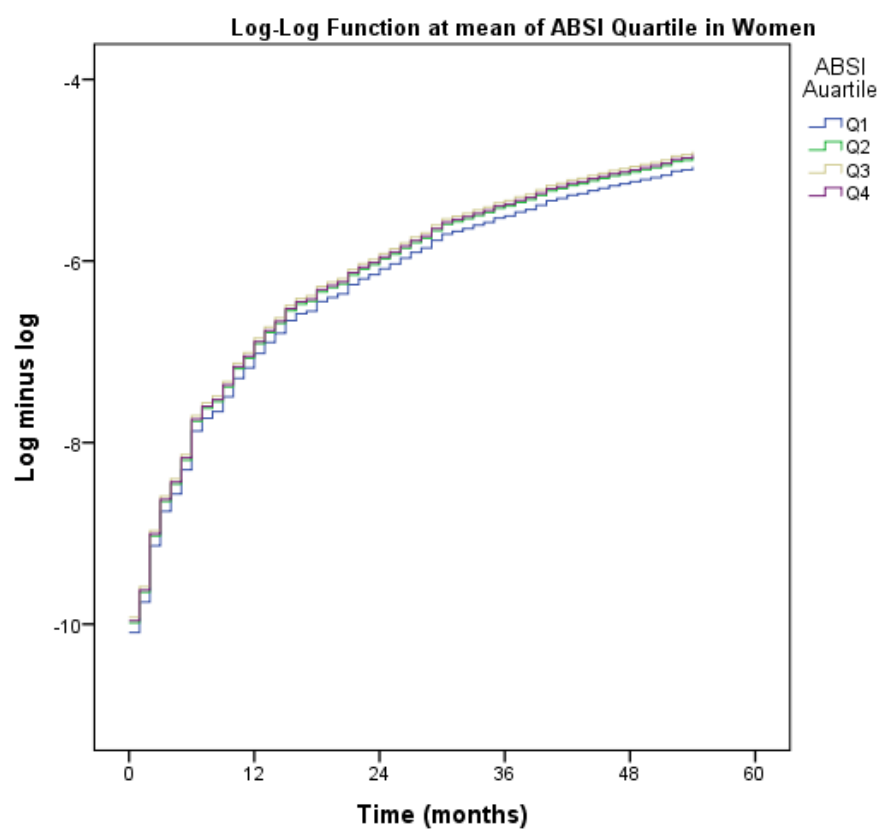

Supplement: S2 Fig — (PDF) [file pone.0177779.s002.pdf]
