# Supplementary material for: Body shape index: Sex-specific differences in predictive power for all-cause mortality in the Japanese population
Source: PLoS One. 2017 May 16;12(5):e0177779. doi: 10.1371/journal.pone.0177779 (PMC5433760; doi:10.1371/journal.pone.0177779)
Supplement: S1 Table — (DOCX) [file pone.0177779.s003.docx]

**S1 Table. Characteristics at enrollment and all-cause mortality over the 4-year follow-up divided by quartile of a body shape index in men**

|  | Q1 | Q2 | Q3 | Q4 | *P*-value |
| --- | --- | --- | --- | --- | --- |
| Number | 16,622 | 16,622 | 16,619 | 16,621 |  |
| Age, years | 58.5 (9.3) | 60.9 (8.4) | 62.2 (7.7) | 63.8 (6.7) | <0.01 |
| ABSI | 0.0759 (0.0020) | 0.0793 (0.0007) | 0.0816 (0.0007) | 0.0851 (0.0020) | <0.01 |
| BMI, kg/m^2^ | 24.1 (3.2) | 24.2 (3.0) | 24.1 (3.0) | 23.8 (3.1) | <0.01 |
| WC, m | 0.81 (0.08) | 0.85 (0.07) | 0.87 (0.07) | 0.90 (0.08) | <0.01 |
| WHtR | 0.49 (0.05) | 0.52 (0.04) | 0.53 (0.04) | 0.55 (0.05) | <0.01 |
| BH, m | 1.65 (0.06) | 1.65 (0.06) | 1.65 (0.06) | 1.65 (0.06) | 0.31 |
| BW, kg | 65.4 (9.9) | 65.7 (9.6) | 65.6 (9.4) | 64.7 (9.6) | <0.01 |
| SBP, mmHg | 128 (17) | 130 (17) | 131 (17) | 132 (17) | <0.01 |
| HDL-C, mg/dL | 57.9 (15.1) | 56.3 (14.8) | 55.7 (14.7) | 55.7 (14.8) | <0.01 |
| HbA1c, % | 5.31 (0.74) | 5.38 (0.80) | 5.41 (0.82) | 5.47 (0.90) | <0.01 |
| eGFR, mL/min/1.73m^2^ | 75.2 (15.4) | 74.5 (15.6) | 74.2 (15.6) | 74.7 (16.7) | <0.01 |
| eGFR categories, n (%) |  |  |  |  | <0.01 |
| G1 | 2,380 (14.3%) | 2,161 (13.0%) | 1,991 (12.0%) | 2,160 (13.0%) |  |
| G2 | 11,717 (70.5%) | 11,598 (69.8%) | 11,658 (70.1%) | 11,529 (69.4%) |  |
| G3a | 2,265 (13.6%) | 2,586 (15.6%) | 2,675 (16.1%) | 2,538 (15.3%) |  |
| G3b | 219 (1.3%) | 245 (1.5%) | 249 (1.5%) | 324 (1.9%) |  |
| G4 | 27 (0.2%) | 22 (0.1%) | 35 (0.2%) | 53 (0.3%) |  |
| G5 | 14 (0.1%) | 10 (0.1%) | 11 (0.1%) | 17 (0.1%) |  |
| Anti-hypertensive drug | 22.2% | 26.9% | 29.9% | 34.0% | <0.01 |
| Anti-diabetes drug | 4.9% | 5.7% | 6.9% | 8.0% | <0.01 |
| Anti-dyslipidemic drug | 7.3% | 8.5% | 9.6% | 10.7% | <0.01 |
| Past history of CVD | 7.7% | 9.0% | 10.1% | 12.3% | <0.01 |
| Current smoking | 26.9% | 26.6% | 27.6% | 29.4% | <0.01 |
| Urine dipstick test, n(%) |  |  |  |  | <0.01 |
| - | 14,041 (84.5%) | 13,982 (84.1%) | 13,818 (83.1%) | 13,512 (81.3%) |  |
| ± | 1,486 (8.9%) | 1,509 (9.1%) | 1,560 (9.4%) | 1,615 (9.7%) |  |
| 1+ | 744 (4.5%) | 725 (4.4%) | 797 (4.8%) | 970 (5.8%) |  |
| 2+ | 255 (1.5%) | 322 (1.9%) | 331 (2.0%) | 396 (2.4%) |  |
| 3+ | 96 (0.6%) | 84 (0.5%) | 113 (0.7%) | 128 (0.8%) |  |
| All-cause mortality, n (%) | 226 (1.4%) | 247 (1.5%) | 329 (2.0%) | 431 (2.6%) | <0.01 |

Data given as mean (standard deviation) unless otherwise specified. P-values refer to the differences between the groups.

Abbreviations: ABSI, a body shape index; BH, body height; BMI, body mass index; BW, body weight; CVD, cardiovascular disease; eGFR, estimated glomerular filtration

Estimated GFR categories defined according to eGFR levels; G1, ≧90 mL/min/1.73m^2^; G2, 60-89 mL/min/1.73m^2^; G3a, 45-59 mL/min/1.73m^2^; G3b 30-44 mL/min/1.73m^2^; G4, 15-29 mL/min/1.73m^2^; G5, <15 mL/min/1.73m^2^
