# Supplementary material for: Body shape index: Sex-specific differences in predictive power for all-cause mortality in the Japanese population
Source: PLoS One. 2017 May 16;12(5):e0177779. doi: 10.1371/journal.pone.0177779 (PMC5433760; doi:10.1371/journal.pone.0177779)
Supplement: S2 Table — (DOCX) [file pone.0177779.s004.docx]

**S2 Table. Characteristics at enrollment and all-cause mortality over the 4-year follow-up divided by quartile of a body shape index in women**

|  | Q1 | Q2 | Q3 | Q4 | *P*-value |
| --- | --- | --- | --- | --- | --- |
| Number | 23,537 | 23,534 | 23,535 | 23,535 |  |
| Age, years | 58.7 (8.7) | 60.9 (7.8) | 62.7 (6.8) | 64.7 (5.6) | <0.01 |
| ABSI | 0.0765 (0.0027) | 0.0818 (0.0011) | 0.0855 (0.0010) | 0.0904 (0.0026) | <0.01 |
| BMI, kg/m^2^ | 22.9 (3.7) | 23.3 (3.6) | 23.4 (3.4) | 22.8 (3.2) | <0.01 |
| WC, m | 0.76 (0.09) | 0.82 (0.08) | 0.86 (0.08) | 0.89 (0.83) | <0.01 |
| WHtR | 0.50 (0.06) | 0.54 (0.06) | 0.57 (0.06) | 0.59 (0.06) | <0.01 |
| BH, m | 1.53 (0.06) | 1.52 (0.06) | 1.52 (0.06) | 1.52 (0.06) | <0.01 |
| BW, kg | 53.5 (9.0) | 54.2 (8.5) | 54.0 (8.1) | 52.3 (7.8) | <0.01 |
| SBP, mmHg | 125 (18) | 127 (18) | 128 (18) | 128 (18) | <0.01 |
| HDL-C, mg/dL | 66.3 (16.1) | 63.9 (15.2) | 63.2 (14.8) | 63.4 (14.8) | <0.01 |
| HbA1c, % | 5.25 (0.55) | 5.32 (0.64) | 5.36 (0.65) | 5.37 (0.65) | <0.01 |
| eGFR, mL/min/1.73m^2^ | 77.4 (16.3) | 76.2 (16.1) | 75.5 (15.8) | 75.7 (16.3) | <0.01 |
| eGFR categories, n (%) |  |  |  |  | <0.01 |
| G1 | 5,485 (23.3%) | 5,362 (22.8%) | 5,406 (23.0%) | 5,898 (25.1%) |  |
| G2 | 15,614 (66.3%) | 15,477 (65.8%) | 15,287 (65.0%) | 14,967 (63.6%) |  |
| G3a | 2,256 (9.6%) | 2,477 (10.5%) | 2,571 (10.9%) | 2,384 (10.1%) |  |
| G3b | 138 (0.6%) | 179 (0.8%) | 217 (0.9%) | 229 (1.0%) |  |
| G4 | 24 (0.1%) | 28 (0.1%) | 36 (0.2%) | 41 (0.2%) |  |
| G5 | 20 (0.1%) | 11 (0.0%) | 18 (0.1%) | 16 (0.1%) |  |
| Anti-hypertensive drug | 19.9% | 23.6% | 26.2% | 26.5% | <0.01 |
| Anti-diabetes drug | 2.6% | 3.6% | 4.0% | 4.2% | <0.01 |
| Anti-dyslipidemic drug | 13.0% | 16.4% | 17.7% | 19.6% | <0.01 |
| Past history of CVD | 4.8% | 5.8% | 6.3% | 7.3% | <0.01 |
| Current smoking | 6.7% | 5.8% | 5.0% | 4.2% | <0.01 |
| Urine dipstick test, n(%) |  |  |  |  | 0.36 |
| - | 21,048 (89.4%) | 21,120 (89.7%) | 21,100 (89.7%) | 21,137 (89.8%) |  |
| ± | 1,593 (6.8%) | 1,567 (6.7%) | 1,565 (6.6%) | 1,539 (6.5%) |  |
| 1+ | 641 (2.7%) | 612 (2.6%) | 619 (2.6%) | 624 (2.7%) |  |
| 2+ | 189 (0.8%) | 168 (0.7%) | 190 (0.8%) | 196 (0.8%) |  |
| 3+ | 66 (0.3%) | 67 (0.3%) | 61 (0.3%) | 39 (0.2%) |  |
| All-cause mortality, n (%) | 126 (0.5%) | 158 (0.7%) | 186 (0.8%) | 198 (0.8%) | <0.01 |

Data given as mean (standard deviation) unless otherwise specified. P-values refer to the differences between the groups.

Abbreviations: ABSI, a body shape index; BH, body height; BMI, body mass index; BW, body weight; CVD, cardiovascular disease; eGFR, estimated glomerular filtration

Estimated GFR categories defined according to eGFR levels; G1, ≧90 mL/min/1.73m^2^; G2, 60-89 mL/min/1.73m^2^; G3a, 45-59 mL/min/1.73m^2^; G3b 30-44 mL/min/1.73m^2^; G4, 15-29 mL/min/1.73m^2^; G5, <15 mL/min/1.73m^2^
