# Supplementary material for: Body shape index: Sex-specific differences in predictive power for all-cause mortality in the Japanese population
Source: PLoS One. 2017 May 16;12(5):e0177779. doi: 10.1371/journal.pone.0177779 (PMC5433760; doi:10.1371/journal.pone.0177779)
Supplement: S3 Table — (DOCX) [file pone.0177779.s005.docx]

**S3 Table. Correlation between anthropometric parameters in men without chronic kidney disease**

|  | ABSI | BMI | WC | WHtR | BH | BW |
| --- | --- | --- | --- | --- | --- | --- |
| ABSI |  | -0.043^**^ | 0.417^**^ | 0.412^**^ | 0.000 | -0.035^**^ |
| BMI | -0.043^**^ |  | 0.840^**^ | 0.844^**^ | -0.018^**^ | 0.835^**^ |
| WC | 0.417^**^ | 0.840^**^ |  | 0.909^**^ | 0.181^**^ | 0.819^**^ |
| WHtR | 0.412^**^ | 0.844^**^ | 0.909^**^ |  | -0.206^**^ | 0.601^**^ |
| BH | 0.000 | -0.018^**^ | 0.181^**^ | -0.206^**^ |  | 0.492^**^ |
| BW | -0.035^**^ | 0.835^**^ | 0.819^**^ | 0.601^**^ | 0.492^**^ |  |

Correlation described in terms of Spearman’s correlation coefficient.

**P*<0.05; ***P*<0.001

Abbreviations: ABSI, a body shape index; BH, body height; BMI, body mass index; BW, body weight; WC, waist circumference; WHtR, waist-to-height ratio.
