# Supplementary material for: Body shape index: Sex-specific differences in predictive power for all-cause mortality in the Japanese population
Source: PLoS One. 2017 May 16;12(5):e0177779. doi: 10.1371/journal.pone.0177779 (PMC5433760; doi:10.1371/journal.pone.0177779)
Supplement: S4 Table — (DOCX) [file pone.0177779.s006.docx]

**S4 Table. Correlation between anthropometric parameters in women without chronic kidney disease**

|  | ABSI | BMI | WC | WHtR | BH | BW |
| --- | --- | --- | --- | --- | --- | --- |
| ABSI |  | 0.002 | 0.539^**^ | 0.538^**^ | -0.083^**^ | -0.040^**^ |
| BMI | 0.002 |  | 0.799^**^ | 0.804^**^ | -0.151^**^ | 0.861^**^ |
| WC | 0.539^**^ | 0.799^**^ |  | 0.947^**^ | -0.017^**^ | 0.748^**^ |
| WHtR | 0.538^**^ | 0.804^**^ | 0.947^**^ |  | -0.308^**^ | 0.599^**^ |
| BH | -0.083^**^ | -0.151^**^ | -0.017^**^ | -0.308^**^ |  | 0.329^**^ |
| BW | -0.040^**^ | 0.861^**^ | 0.748^**^ | 0.599^**^ | 0.329^**^ |  |

Correlation described in terms of Spearman’s correlation coefficient.

**P*<0.05; ***P*<0.001

Abbreviations: ABSI, a body shape index; BH, body height; BMI, body mass index; BW, body weight; WC, waist circumference; WHtR, waist-to-height ratio.
