# Supplementary material for: Body shape index: Sex-specific differences in predictive power for all-cause mortality in the Japanese population
Source: PLoS One. 2017 May 16;12(5):e0177779. doi: 10.1371/journal.pone.0177779 (PMC5433760; doi:10.1371/journal.pone.0177779)
Supplement: S5 Table — (DOCX) [file pone.0177779.s007.docx]

**S5 Table. Correlation between anthropometric parameters in men with chronic kidney disease**

|  | ABSI | BMI | WC | WHtR | BH | BW |
| --- | --- | --- | --- | --- | --- | --- |
| ABSI |  | -0.048^**^ | 0.423^**^ | 0.407^**^ | 0.025^**^ | -0.023^**^ |
| BMI | -0.048^**^ |  | 0.833^**^ | 0.843^**^ | -0.039^**^ | 0.831^**^ |
| WC | 0.423^**^ | 0.833^**^ |  | 0.906^**^ | 0.176^**^ | 0.820^**^ |
| WHtR | 0.407^**^ | 0.843^**^ | 0.906^**^ |  | -0.213^**^ | 0.602^**^ |
| BH | 0.025^**^ | -0.039^**^ | 0.176^**^ | -0.213^**^ |  | 0.478^**^ |
| BW | -0.023^**^ | 0.831^**^ | 0.820^**^ | 0.602^**^ | 0.478^**^ |  |

Correlation described in terms of Spearman’s correlation coefficient.

**P*<0.05; ***P*<0.001

Abbreviations: ABSI, a body shape index; BH, body height; BMI, body mass index; BW, body weight; WC, waist circumference; WHtR, waist-to-height ratio.
