# Supplementary material for: Body shape index: Sex-specific differences in predictive power for all-cause mortality in the Japanese population
Source: PLoS One. 2017 May 16;12(5):e0177779. doi: 10.1371/journal.pone.0177779 (PMC5433760; doi:10.1371/journal.pone.0177779)
Supplement: S6 Table — (DOCX) [file pone.0177779.s008.docx]

**S6 Table. Correlation between anthropometric parameters in women with chronic kidney disease**

|  | ABSI | BMI | WC | WHtR | BH | BW |
| --- | --- | --- | --- | --- | --- | --- |
| ABSI |  | -0.021^*^ | 0.495^**^ | 0.483^**^ | -0.051^**^ | -0.044^**^ |
| BMI | -0.021^*^ |  | 0.814^**^ | 0.825^**^ | -0.173^**^ | 0.872^**^ |
| WC | 0.495^**^ | 0.814^**^ |  | 0.947^**^ | -0.024^**^ | 0.777^**^ |
| WHtR | 0.483^**^ | 0.825^**^ | 0.947^**^ |  | -0.312^**^ | 0.639^**^ |
| BH | -0.051^**^ | -0.173^**^ | -0.024^**^ | -0.312^**^ |  | 0.286^**^ |
| BW | -0.044^**^ | 0.872^**^ | 0.777^**^ | 0.639^**^ | 0.286^**^ |  |

Correlation described in terms of Spearman’s correlation coefficient.

**P*<0.05; ***P*<0.001

Abbreviations: ABSI, a body shape index; BH, body height; BMI, body mass index; BW, body weight; WC, waist circumference; WHtR, waist-to-height ratio.
