# Supplementary material for: Body shape index: Sex-specific differences in predictive power for all-cause mortality in the Japanese population
Source: PLoS One. 2017 May 16;12(5):e0177779. doi: 10.1371/journal.pone.0177779 (PMC5433760; doi:10.1371/journal.pone.0177779)
Supplement: S7 Table — (DOCX) [file pone.0177779.s009.docx]

**S7 Table. Hazard ratios and 95% confidence intervals of other covariates when risk of 1-SD increase of ABSI for all-cause mortality was analyzed by Cox analysis**

|  |  | Men | | | | Women | | | |
| --- | --- | --- | --- | --- | --- | --- | --- | --- | --- |
|  |  | non-CKD cohort | | CKD cohort | | non-CKD cohort | | CKD cohort | |
|  |  | HR (95%CI) | *P*-value | HR (95%CI) | *P*-value | HR (95%CI) | *P*-value | HR (95%CI) | *P*-value |
| Age (+1SD) | | 1.52 (1.39 to 1.65) | <0.01 | 1.42 (1.20 to 1.68) | <0.01 | 1.61 (1.42 to 1.82) | <0.01 | 1.26 (0.99 to 1.60) | 0.06 |
| SBP (+1SD) | | 1.10 (1.02 to 1.18) | 0.01 | 0.97 (0.87 to 1.07) | 0.51 | 1.11 (1.01 to 1.22) | 0.03 | 1.20 (1.04 to 1.39) | 0.01 |
| HDL (+1SD) | | 0.96 (0.89 to 1.03) | 0.26 | 1.00 (0.89 to 1.12) | 1.00 | 0.93 (0.84 to 1.02) | 0.12 | 0.88 (0.75 to 1.05) | 0.15 |
| A1c (+1SD) | | 1.07 (1.01 to 1.13) | 0.02 | 1.13 (1.05 to 1.21) | <0.01 | 1.03 (0.93 to 1.15) | 0.54 | 1.09 (0.95 to 1.24) | 0.23 |
| eGFR (+1SD) | | 1.14 (1.05 to 1.23) | <0.01 | 0.89 (0.78 to 1.01) | 0.07 | 1.15 (1.08 to 1.23) | <0.01 | 0.86 (0.71 to 1.05) | 0.13 |
| antiHTN drug | | 1.08 (0.92 to 1.26) | 0.36 | 1.06 (0.85 to 1.33) | 0.59 | 1.18 (0.96 to 1.45) | 0.12 | 1.27 (0.91 to 1.77) | 0.17 |
| antiDM drug | | 1.25 (0.96 to 1.62) | 0.10 | 1.47 (1.07 to 2.03) | 0.02 | 1.13 (0.71 to 1.82) | 0.61 | 1.35 (0.74 to 2.45) | 0.33 |
| antiDyslipidema drug | | 0.82 (0.63 to 1.06) | 0.13 | 0.81 (0.59 to 1.10) | 0.18 | 0.73 (0.57 to 0.95) | 0.02 | 0.74 (0.51 to 1.09) | 0.13 |
| past CVD | | 1.38 (1.12 to 1.70) | <0.01 | 1.36 (1.05 to 1.77) | 0.02 | 1.09 (0.77 to 1.55) | 0.63 | 2.12 (1.42 to 3.16) | <0.01 |
| current smoking | | 1.70 (1.48 to 1.97) | <0.01 | 1.60 (1.27 to 2.02) | <0.01 | 1.84 (1.30 to 2.60) | <0.01 | 1.44 (0.73 to 2.86) | 0.29 |

Abbreviations: ABSI, a body shape index; SBP, systolic blood pressure; HDL, high density lipoprotein; A1c, glycated hemoglobin; eGFR, estimated glomerular filtration rate; HTN, hypertension; DM, diabetes mellitus; CVD, cardiovascular disease; CKD, chronic kidney disease.
