# Supplementary material for: Body shape index: Sex-specific differences in predictive power for all-cause mortality in the Japanese population
Source: PLoS One. 2017 May 16;12(5):e0177779. doi: 10.1371/journal.pone.0177779 (PMC5433760; doi:10.1371/journal.pone.0177779)
Supplement: S8 Table — (DOCX) [file pone.0177779.s010.docx]

**S8 Table. Risk for all-cause mortality in participants with and without chronic kidney disease**

|  |  |  |  | Q1 | Q2 | | Q3 | | Q4 | |
| --- | --- | --- | --- | --- | --- | --- | --- | --- | --- | --- |
|  |  |  |  |  | HR (95%CI) | *P*-value | HR (95%CI) | *P*-value | HR (95%CI) | *P*-value |
| Men | non-CKD | BMI | Model 1 | 1.00 (Ref) | 0.76 (0.64 to 0.91) | <0.01 | 0.71 (0.58 to 0.85) | <0.01 | 0.82 (0.68 to 0.98) | 0.03 |
|  |  |  | Model 2 | 1.00 (Ref) | 0.76 (0.63 to 0.91) | <0.01 | 0.69 (0.57 to 0.84) | <0.01 | 0.75 (0.61 to 0.91) | <0.01 |
|  |  | WC | Model 1 | 1.00 (Ref) | 0.78 (0.65 to 0.95) | 0.01 | 0.82 (0.68 to 1.00) | 0.045 | 1.07 (0.90 to 1.28) | 0.45 |
|  |  |  | Model 2 | 1.00 (Ref) | 0.78 (0.65 to 0.95) | 0.01 | 0.80 (0.66 to 0.97) | 0.02 | 0.98 (0.81 to 1.19) | 0.86 |
|  |  | WHtR | Model 1 | 1.00 (Ref) | 0.96 (0.80 to 1.15) | 0.69 | 0.80 (0.66 to 0.96) | 0.02 | 0.76 (0.63 to 0.92) | 0.01 |
|  |  |  | Model 2 | 1.00 (Ref) | 0.82 (0.67 to 0.99) | 0.04 | 0.75 (0.62 to 0.92) | 0.01 | 0.95 (0.78 to 1.15) | 0.58 |
|  | CKD | BMI | Model 1 | 1.00 (Ref) | 0.60 (0.45 to 0.80) | <0.01 | 0.50 (0.37 to 0.67) | <0.01 | 0.70 (0.54 to 0.91) | 0.01 |
|  |  |  | Model 2 | 1.00 (Ref) | 0.60 (0.44 to 0.80) | <0.01 | 0.49 (0.36 to 0.66) | <0.01 | 0.64 (0.48 to 0.85) | <0.01 |
|  |  | WC | Model 1 | 1.00 (Ref) | 0.62 (0.46 to 0.84) | <0.01 | 0.76 (0.57 to 1.00) | 0.05 | 0.74 (0.56 to 0.97) | 0.03 |
|  |  |  | Model 2 | 1.00 (Ref) | 0.62 (0.45 to 0.83) | <0.01 | 0.72 (0.54 to 0.96) | 0.02 | 0.66 (0.49 to 0.89) | 0.01 |
|  |  | WHtR | Model 1 | 1.00 (Ref) | 1.22 (0.93 to 1.60) | 0.16 | 0.78 (0.59 to 1.04) | 0.09 | 0.77 (0.58 to 1.01) | 0.06 |
|  |  |  | Model 2 | 1.00 (Ref) | 0.64 (0.46 to 0.87) | 0.01 | 0.60 (0.44 to 0.82) | <0.01 | 0.75 (0.56 to 1.00) | 0.048 |
| Women | non-CKD | BMI | Model 1 | 1.00 (Ref) | 0.78 (0.61 to 1.00) | 0.05 | 0.85 (0.67 to 1.08) | 0.85 | 0.92 (0.72 to 1.17) | 0.50 |
|  |  |  | Model 2 | 1.00 (Ref) | 0.75 (0.58 to 0.97) | 0.03 | 0.78 (0.61 to 1.00) | 0.05 | 0.78 (060 to 1.01) | 0.06 |
|  |  | WC | Model 1 | 1.00 (Ref) | 0.74 (0.57 to 0.96) | 0.02 | 0.82 (0.64 to 1.05) | 0.11 | 0.96 (0.76 to 1.21) | 0.72 |
|  |  |  | Model 2 | 1.00 (Ref) | 0.70 (0.54 to 0.90) | 0.01 | 0.75 (0.58 to 0.97) | 0.03 | 0.82 (0.64 to 1.06) | 0.12 |
|  |  | WHtR | Model 1 | 1.00 (Ref) | 0.91 (0.70 to 1.18) | 0.45 | 0.92 (0.71 to 1.19) | 0.53 | 1.03 (0.80 to 1.32) | 0.84 |
|  |  |  | Model 2 | 1.00 (Ref) | 0.87 (0.67 to 1.13) | 0.28 | 0.84 (0.65 to 1.10) | 0.20 | 0.88 (0.67 to 1.15) | 0.35 |
|  | CKD | BMI | Model 1 | 1.00 (Ref) | 1.03 (0.62 to 1.71) | 0.91 | 1.10 (0.68 to 1.79) | 0.69 | 1.41 (0.90 to 2.21) | 0.14 |
|  |  |  | Model 2 | 1.00 (Ref) | 0.95 (0.57 to 1.59) | 0.85 | 0.94 (0.58 to 1.54) | 0.81 | 1.03 (0.63 to 1.67) | 0.91 |
|  |  | WC | Model 1 | 1.00 (Ref) | 1.16 (0.72 to 1.85) | 0.54 | 0.86 (0.53 to 1.40) | 0.54 | 1.24 (0.80 to 1.92) | 0.34 |
|  |  |  | Model 2 | 1.00 (Ref) | 1.05 (0.65 to 1.68) | 0.85 | 0.72 (0.44 to 1.19) | 0.20 | 0.89 (0.56 to 1.42) | 0.63 |
|  |  | WHtR | Model 1 | 1.00 (Ref) | 1.04 (0.62 to 1.76) | 0.88 | 1.33 (0.82 to 2.17) | 0.25 | 1.36 (0.85 to 2.18) | 0.20 |
|  |  |  | Model 2 | 1.00 (Ref) | 0.93 (0.54 to 1.57) | 0.77 | 1.10 (0.67 to 1.82) | 0.71 | 0.98 (0.59 to 1.63) | 0.95 |

Risk was assessed using Cox regression analysis. Model 1, was adjusted for age. Model 2 consisted of Model 1 + adjustments for systolic blood pressure (+1SD), high-density lipoprotein cholesterol (+1SD), glycated hemoglobin (+1SD), estimated glomerular filtration rate (+1SD), drug use (anti-hypertension, anti-diabetes, or anti-dyslipidemia), past history of cardiovascular disease, and current smoking status (smoker).

Data given as hazard ratio (HR) with 95% confidence interval (95%CI) and stratified by quartiles (Q1 to Q4) for the distribution of values for each anthropometric parameter.

Abbreviations: BMI, body mass index; CKD, chronic kidney disease; Ref, reference; WC, waist circumference; WHtR, waist-to-height ratio.
